# Evaluating the Significance of Obesity or Excessive Weight in Various Mental Health Disorders: A Systematic Review

**DOI:** 10.7759/cureus.78251

**Published:** 2025-01-30

**Authors:** Taiwo A Falaiye, Okelue E Okobi, Chidimma I Oramu, Anuoluwapo O Jegede

**Affiliations:** 1 Family Medicine, Northwest Health LaPorte, La Porte, USA; 2 Family Medicine, Medficient Health Systems, Laurel, USA; 3 Family Medicine, Lakeside Medical Center, Belle Glade, USA; 4 Family Medicine, Larkin Community Hospital Palm Springs Campus, Miami, USA; 5 Psychiatry and Behavioral Sciences, Mildmay Oaks Priory Hospital, Hampshire, GBR; 6 Community Medicine, Danylo Halytsky Lviv Medical University, Lviv, UKR

**Keywords:** body mass index, mental health disorders, metabolic syndrome, obesity, weight gain

## Abstract

Obesity is a major global public health challenge affecting all countries and communities. The link between obesity and various types of physical morbidities has been widely acknowledged in different studies. Despite the immense impact of obesity on mental health, its full effect on the areas has not been as explored as the impact on physical health has been. Following an extensive review of various recent studies, the objective of this study is to evaluate the correlations and effects of obesity on mental health disorders, in addition to reflecting on the significance of assessing the correlation. The other objective of this study is to evaluate obesity prevalence in mentally ill individuals. We believe that the realization of these objectives will address the existing literature gaps within the population of mentally ill persons in addition to aiding with the necessary preventive knowledge that will enable the provision of optimum mental and physical health. Therefore, this study entailed the performance of a systematic review of several online databases, including Scopus, Web of Sciences, PubMed, Google Scholar, and MEDLINE. This systematic review also utilized an increasingly robust methodology based on the Cochrane guidelines and Preferred Reporting Items for Systematic Reviews and Meta-Analyses (PRISMA) guidelines. Thus, the inclusion criteria stipulated that only studies published between 2010 and 2024 and published in the English language were to be included in this systematic review. The quality of the included studies was assessed using an appraisal tool for cross-sectional studies. As a result, 12 studies met the inclusion criteria for this systematic review and were reviewed. The findings indicate that the prevalence rate of obesity in individuals with mental health disorders surpasses the prevalence of the condition within the general population, suggesting that mentally ill persons are at a higher risk of developing obesity, even as one of the major side effects of psychiatric treatment is excessive weight gain. Individuals with personality disorders, including borderline personality disorder, avoidant personality disorder, dependent personality disorder, and antisocial personality disorder, among others, presented the highest prevalence rate of obesity compared to those with psychosis. Prospective studies should focus on evaluating the various mitigating factors that underlie the weight gain and obesity development that occur across mental health disorders.

## Introduction and background

At present, obesity is considered a significant global public health concern affecting nearly all nations and communities across the globe. Being a chronic condition, obesity is mainly caused by the intricate interplay of various factors, including genetics, environmental, behavioral, psychosocial, and neuroendocrinological factors [1]. A correlation has been found between obesity and different types of physical illnesses, disabilities, and secondary complications [2]. Moreover, an adverse effect of obesity on mental health has been acknowledged, even though this has not been sufficiently studied, regardless of the observation that there is increased awareness concerning obesity and its effects on individuals’ physical health. Effective management of obesity is a significant challenge requiring psychological interventions to tackle the different related psychological effects and offer support to obese persons facing challenges in adopting weight management interventions [1-3]. Therefore, it is vital to explore the extant correlations between obesity and mental health and the treatment interventions for effectual support.

Obesity prevalence

It is estimated that, as of 2024, over 1 billion individuals are living with obesity, including approximately 159 million children and adolescents aged between 5 and 19 years and 880 million adults [3]. Regardless of the existing variations, obesity prevalence rates have significantly increased over the decades. For instance, in children and adolescents aged between 5 and 19 years, obesity prevalence is estimated to have risen 10-fold from 4% in 1975 to nearly 20% as of 2022 [3]. According to the World Obesity Federation, in 1975, only 0.6% of girls aged 5-19 years had obesity compared to 6.9% in 2022, even as only 0.9% of boys had obesity in 1975 compared to 9.3% in 2022 [3]. This observation implies that nearly 94 million boys and 65 million girls had obesity as of 2022, in comparison to 6 million boys and 5 million girls in 1975. Consequently, in adult persons, obesity prevalence has increased 4-fold in men from 3% in 1975 to 14% in 2022 and 3-fold in women from 6.6% in 1975 to 18.5% in 2022 [3]. The implication is that, as of 2022, nearly 374 million men and 504 million women were obese.

Moreover, across the globe, recent studies have indicated that nearly 60% of individuals residing in Europe were either obese or overweight [4]. For instance, in the United Kingdom, approximately 30% of adults were categorized as obese. In comparison, nearly 38% were considered overweight in a study conducted in 2021 [4], even though it was indicated that men (68.6%) are at higher risk of developing obesity as compared to women (59%) [4]. Further, in India, recent studies have disclosed that the prevalence of obesity and central obesity has significantly increased between 11.8% to 31.3% and between 16.9% and 36.3%, concurrently [5]. Similarly, in the United States, obesity has been acknowledged as being widespread within the clinical population, given that more than three-quarters of the clinic population was considered either obese or overweight, with only half receiving an obesity diagnosis [6].

Obesity and mental illness

Over time, significant focus and attention have been directed toward the poor physical health of persons with mental illness, with the population having a mortality rate that is almost three times higher as compared to the general population [7,8]. Even though ischemic heart disease remains the most significant contributor to the mortality rates within the mentally ill populace, considerable correlations have been reported between obesity, type 2 diabetes, and cardiovascular disease, with the most prevalent lifestyle factors among the mentally ill persons being consumption of highly saturated fat diets and lack of physical exercise [7]. In this regard, Correll et al. have observed that the risk of developing obesity is over four times greater in persons with schizophrenia and nearly two times greater in mentally ill persons with bipolar disorder (BD) and major depressive disorder (MDD) in comparison to the general population [8]. Further, Vancampfort et al. have asserted that, in general, the percentage of mentally ill persons with abdominal obesity is approximately 50-63%, reliant on the evaluation criterion used [9]. In such mentally ill persons, lifestyle factors, including increased consumption of unhealthy diets, physical inactivity, and the use of antipsychotic drugs, have been acknowledged to significantly contribute to their development of obesity [10].

Still, in a recent study conducted by Porter and Evans, the findings disclosed that mentally ill persons had lower physical activity rates and a higher rate of poor dietary habits in comparison to the general population [11]. However, varying results have been arrived at in a study conducted by Osborn et al., who disclosed that in comparison to a matched sample of individuals diagnosed with psychotic disorders, similar levels of consumption of the total unsaturated and saturated fats were reported between the two study groups [12]. The findings of the study are comparable to those of a recent survey conducted in New Zealand, despite the latter disclosing that the level of sugar consumption was significantly higher in mentally ill persons, especially those with psychotic illnesses [13]. Moreover, research has further disclosed significant disruption of metabolism attributable to the use of various psychotic medications. For instance, a number of studies have focused on the evaluation of weight gain as a result of the use of antipsychotics and both typical and atypical mood-stabilizing medications [14,15]. In this regard, the use of various antidepressant drugs, including tricyclics, has been reported to lead to significant weight gain [15], and this has been further attributed to the use of selective serotonin reuptake inhibitors (SSRIs). Weight gain due to antipsychotics has been attributed to an increase in food intake and appetite and delays in satiety signaling. Consequently, antagonism at the histaminergic H1 and serotoninergic 5-HT2C receptors have been reported as the main mechanisms that add to such side effects. Antidepressants like olanzapine and clozapine have the greatest weight gain liability and this has been attributed to their sturdy binding affinity to the H1 and 5-HT2C receptors [16]. In most instances, studies have identified H1 antagonism as a robust predictor of weight gain [17]. The decrement in the level of caloric expenditure resulting from the effects of sedatives, along with a subsequent increment in the consumption of caloric beverages resulting from thirst, and dry throat and mouth, induced by the use of specific antipsychotics significantly contribute to antipsychotic-induced weight gain.

In their study, Mazereel et al. assessed epidemiological data regarding the correlation between the level and incidence of weight gain and the use of antidepressants, antipsychotics, and mood stabilizers in mentally ill persons [18]. The study disclosed that there was significant weight gain with the use of antipsychotics (typical and atypical), tricyclic antidepressants, and mood stabilizers. At present, data on the correlations between SSRI use and weight effects remain limited. However, from the available data, it has been disclosed that even though SSRIs lead to weight loss during the initial few weeks of use, they lead to considerable weight gain in the long run [19].

Several hypotheses have been formulated to explain the bidirectional correlations between obesity and mental disorders, including the systematic inflammation hypothesis, the hypothalamic-pituitary-adrenal (HPA) axis hypothesis, the self-medication hypothesis, and the maladaptive coping hypothesis. Regarding the systemic inflammation hypotheses, several studies have disclosed that nutritious diets can reduce the inflammatory processes that occur in the brain [20,21]. Diets rich in calories and with higher sugar and saturated fats have, on the other hand, been disclosed to stimulate immune activation while simultaneously affecting cognitive processes, including hippocampal functions [22,23]. Also, diets with high saturated fats and carbohydrate content have further been disclosed to have reduction effects on the brain-derived neurotrophic factor (BDNF) found within the hippocampus, which further leads to a significant impairment of spatial memory alongside the increased risk of developing depression [20,24]. Adipocytes initiate the proinflammatory/cytokines release, including C-reactive proteins, interleukin-2, and interleukin-6, known to induce psychological stress [25]. In this regard, various longitudinal studies have disclosed that persons with lower Dietary Inflammatory Index scores tend to experience depression incidences, whose symptoms may be alleviated through the use of anti-inflammatory medications [26,27]. Such findings have indicated an existing relationship between the type and quality of diets, severe inflammation, and obesity/mental health outcomes.

Further, the hypothalamic-pituitary-adrenal (HPA) axis hypothesis has clarified the bi-directional correlation between obesity and mental disorders, with correlations between obesity and mental disorders such as depression having been observed to involve HPA axis dysregulation. For instance, persons with mental disorders have been acknowledged to have severely elevated cortisol levels that increase HPA axis dysfunction probability [28]. HPA overstimulation eventually results in the reduction in the sensitivity of glucocorticoid receptors, alongside ensuing insufficient negative feedback mechanisms, which leads to continuous stress hormone production, which, in turn, increases obesity development risk [25]. Further, the HPA axis hypothesis is critical in younger persons, given that previous studies have established that cortisol levels tend to vary based on age and also influence depression vulnerability [29,30].

Also, regarding the self-medication hypothesis and maladaptive coping, several studies have disclosed that the “self-medication” hypothesis plays a significant role in connecting mental disorders and obesity [20,31]. Thus, the theory maintains that unhealthy foods offer momentary relief from stress, thereby enhancing the probability of an individual developing a subtle addiction [31]. This may lead to poor dietary habits and an increased probability of obesity and concurrent depression [20]. Additionally, obese experience increased risks of substance use and abuse as a means of coping with negative emotions [32]. The psychosocial stressors might also contribute to various maladaptive coping mechanisms that include the affected persons developing suboptimal sleep patterns [33-35]. Thus, poor sleep patterns and quality are associated with an increased risk of obesity and depression, even as such stress-linked disruptions to sleeping patterns have been acknowledged to play important roles in individuals’ mental well-being [36-38].

Still, several studies reviewed have disclosed that obesity strikingly increased in persons experiencing mental illnesses, including psychological distress, and the resultant metabolic syndrome has become a key concern amongst mentally ill persons [16,18,39]. According to Swarup et al., metabolic syndrome refers to the cluster of various risk factors, including hyperglycemia, dyslipidemia, hypertension, and central obesity, which increases the propensity of an individual to develop type 2 diabetes and cardiovascular diseases [40]. In evaluating the prevalence of metabolic syndrome in persons with mental illnesses, John et al. disclosed that persons without metabolic syndrome diagnosis presented an average BMI of 26.1 and were categorized as overweight, even as individuals who had metabolic syndrome diagnosis presented an average BMI of 32.5 leading to their categorization as obese. In this regard, the main objective of the study was to evaluate the different mental health disorders, including bipolar disorders and schizophrenia, in which the patients were treated using antipsychotic drugs, and their relationship to becoming overweight and obese. Significant correlations have also been disclosed between increments in BMI levels and mental health disorders, particularly depression [41]. For instance, the study by Robinson et al. disclosed that there was a significant correlation between being overweight and obesity, and all mood disorders and certain types of anxiety disorders [42].

Additionally, hormonal and metabolic differences in relation to obesity have been assessed through comparison between mentally ill persons and the general population. Also, Tek et al. conducted a study comparing 71 obese persons diagnosed with schizophrenia and treated using clozapine, and another 50 obese persons of comparable ages but without any mental health disorders [43]. The study disclosed that, although the schizophrenia patients had a faintly lower BMI (30.1) compared to those without the disease, they had a greater waist-to-hip ratio (0.92) compared to those without the disease (0.89), and also that they had a higher girth measurement (98.9) compared to those without the disease (96.9) [43]. Additionally, schizophrenia patients have been observed to have abnormal lipid profiles, impaired homeostasis of glucose-insulin, and hormonal changes within the GH-IGFIGFBP (growth hormone (GH)-insulin-like growth factor (IGF)-IGF binding protein (IGFBP)) axes [44].

Given the significant effects of being overweight and obesity on physical health and quality of life, the existing correlations between obesity and mental health disorders must be explored. While a larger number of studies have mainly focused on the correlations between obesity and depression, there is limited information regarding the correlations between obesity and other mental health disorders [43-46]. To a larger extent, effective management of obesity calls for adequate psychological interventions. However, owing to the fast-changing epidemiological patterns of increased obesity prevalence, there is an urgent need to evaluate the multifaceted interface between obesity and various psychiatric/mental health disorders [44-47]. This systematic review seeks to evaluate the correlations and effects of obesity on mental health disorders, in addition to reflecting on the significance of assessing the correlation and effects of obesity on mental health.

## Review

Materials and methods

For this study, an extensive literature search was conducted on various virtual databases, including PubMed, Web of Sciences, Scopus, Google Scholar, and Embase, for studies published between 2010 and 2024. The studies selected for this systematic review included epidemiological studies, prospective cohort studies, systematic reviews, multicenter studies, and health assessment studies. Duplicate data sources were mainly identified by comparing studies from the same population years. Also, various MeSH keywords were utilized in the search, including Obesity, Mental Health Disorders, and Antipsychotics. The literature search conducted yielded a total of 819 articles.

Inclusion and exclusion criteria

The removal of all duplicate studies was followed by the selection of pertinent articles, which was done based on a three-stage process. The initial stage involved screening the studies’ titles and abstracts while the second stage excluded all articles deemed irrelevant to the study. The third stage entailed performing a comprehensive full-text assessment of each study to confirm its relevance. In this regard, three independent reviewers screened the studies, and potential disagreements were resolved using consensus and discussions.

For this systematic review, the inclusion criteria mainly targeted original studies, including prospective cohort studies, randomized controlled trials (RCTs), and crossover studies, among others that satisfied the following criteria: studies published between 2020 and 2024, original scientific research published in reputable and peer-reviewed journals, full-text articles, and studies initially published in the English language. To further qualify, the included studies had to focus on assessing the correlation between overweight and obesity and various mental health disorders.

Consequently, this study's exclusion criteria included opinion pieces, sponsored clinical trials, editorials, narrative reviews, and studies lacking relevance to the target populations. Irrelevant and inaccessible studies and studies with unsound methodologies were additionally excluded, leading to the elimination of 817 articles.

The data extracted from the included studies for this systematic review included general study attributes, such as sampling methods, authors, and publication year; demographic attributes, including the sample size, age, gender, race, and follow-ups; and data pertaining to interventions, the duration of the intervention, and the measurement method’s weight. Systematic recording of the main findings of every study was also done.

Results

**Figure 1:**
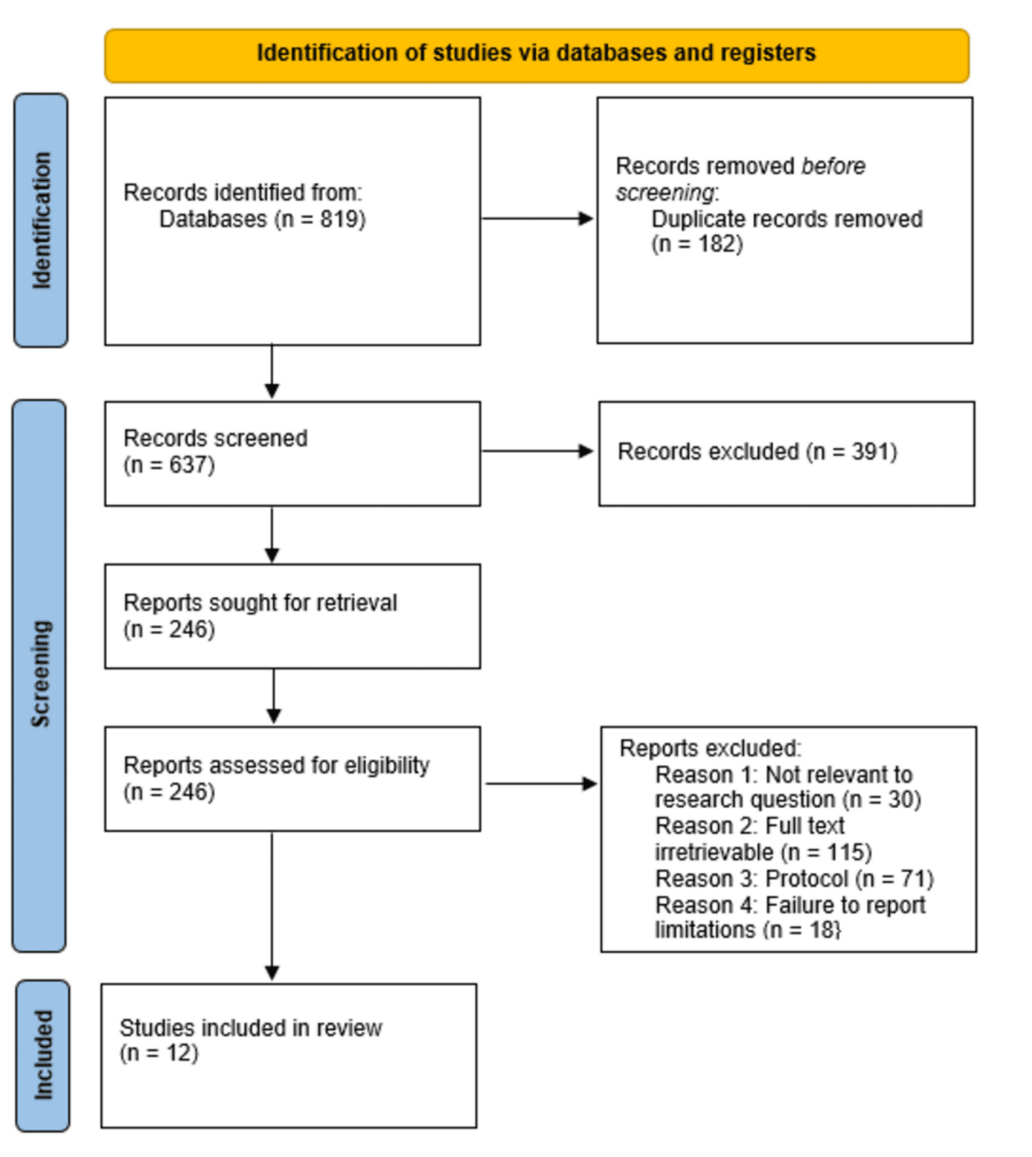
PRISMA flow diagram indicating the study selection process for this systematic review PRISMA: Preferred Reporting Items for Systematic Reviews and Meta-Analyses n: Number of studies/articles

For this systematic review, study selection and inclusion adhered to Preferred Reporting Items for Systematic Reviews and Meta-Analyses (PRISMA) guidelines, and the initial in-depth database search yielded 819 studies. Following the screening, 182 duplicates were excluded. This was followed by screening the studies’ titles and abstracts, which led to the automatic exclusion of 391 ineligible studies. The remaining 246 studies were sought for retrieval and subsequently assessed for eligibility. As a result, 234 studies were excluded for several reasons, including irrelevant research questions (30 studies), irretrievable full texts (115 studies), protocol (71 studies), and failure to report limitations (18 studies). Eventually, 12 studies met the inclusion criteria and have been included in this systematic review, and have been evaluated and discussed alongside findings of other studies corroborating this study's findings [48-76]. The PRISMA flow diagram outlining the article selection process for this study is shown in Figure 1 below.

A summary of the studies included in this systematic review and their findings have been presented in Table 1 below.

**Table 1:** Summary of the studies included in this systematic review

Study/citation	Study design	Sample Size	Findings
Rindler GA, Gries A, Freidl W [48]	Retrospective observational study	16,184 adults aged 50 years and above	Obesity was significantly associated with the increase in the prevalence rates of depression and anxiety in elderly persons. A stronger correlation was observed in females than in males.
Stabouli S, Erdine S, Suurorg L, Jankauskienė A, Lurbe E [49]	Meta-analysis	41 studies with children and young adult participants	The study identified an extant bidirectional correlation between obesity and different eating disorders in younger persons, stressing the importance of early intervention and various psychosocial aspects in mitigating the risks associated with the development of either condition and one exacerbating the other.
Ma J, Xiao L [50]	Cross-sectional study	2,693 women from the National Health and Nutritional Examination Survey (NHANES) data	The study disclosed that obesity was significantly related to moderate-to-severe depressive symptoms in US women, even after adjusting for potential confounders.
Ishii H, Yamada H, Sato R, et al. [51]	Multicenter questionnaire survey	1,276 psychiatric outpatients	The study found that obesity was associated with specific psychiatric diagnoses, including mood and anxiety disorders, with medication use and lifestyle factors as the main contributing factors.
Mitchell AJ, Vancampfort D, Sweers K, et al. [52]	Systematic review and meta-analysis	52 studies comprising 10,055 participants with schizophrenia and related disorders	The study has disclosed that persons with schizophrenia and associated disorders presented a higher prevalence rate of metabolic syndrome and that obesity was a major contributing factor.
Kivimäki M, Jokela M, Hamer M, et al. [53]	Longitudinal cohort study	7,877 participants	The study has disclosed the existence of a bidirectional relationship between obesity and various mental disorders, with available evidence indicating that mental disorders often result in weight gain and vice versa.
Choong E, Bondolfi G, Etter M, et al. [61]	Observational study	515 psychiatric patients	There is a sturdy correlation between psychotropic medications and weight gain and metabolic complications, which necessitates cautious monitoring of the psychiatric populaces.
Kalarchian MA, King WC, Devlin MJ, et al. [62]	Prospective cohort study	2,458 bariatric surgery patients	In assessing the correlation between psychiatric disorders and weight changes in bariatric surgery patients, the study has disclosed that individuals with psychiatric disorders presented different weight loss outcomes, with certain disorders adversely impacting optimal weight reduction.
Lazzari C, Shoka A, Nusair A, Rabottini M [64]	Longitudinal and cross-sectional analysis	239 psychiatric inpatients	The study disclosed that obesity was prevalent in adult psychiatric inpatients, and this was attributable to the effects of psychotropic medications alongside lifestyle factors that lead to weight changes in the study population.
Senra H, Gaglianone CG, McPherson S, Unterrainer H [66]	Meta-analysis	20 studies comprising 2945 binge eating disorder patients	The study disclosed a higher prevalence of personality disorders in adults diagnosed with eating disorders. The study has further identified personality traits along with disorders as the major factors that influence obesity-related behaviors.
Lin HY, Huang CK, Tai CM, et al. [67]	Cross-sectional study	343 patients seeking obesity treatment	The study has disclosed that 42% of participants had, at the minimum, one mental disorder, with anxiety, eating, and mood disorders being the most prevalent. Further, the study disclosed that females were more prone to develop eating and mood disorders than males, even as the surgical group portrayed higher rates of binge eating, adjustment, and sleep disorders.
Nayerifard R, Zahiroddin A, Namjoo M, Rajezi S [76]	Cross-sectional study	204 patients with schizophrenia and bipolar I disorders	The study found that individuals with schizophrenia had a higher prevalence rate of metabolic syndrome than individuals diagnosed with bipolar I disorder. Further, the study findings have stressed the metabolic risks linked to severe mental illnesses.

Quality assessment

The appraisal of the included studies was performed through the use of AXIS (Appraisal tool for Cross-Sectional Studies), a critical appraisal tool with 20 items for studies [45]. In this regard, every included study was assessed by three independent reviewers, with potential disagreements being resolved through group discussions and consensus. Also, every included study was scored 1 (yes) or 0 (no), and “don’t know” for the inapplicable items correspondingly. In general, the quality of the included studies was moderate to high quality, with only four studies being of moderate quality and the rest being of high quality.

Data extraction

The authors used a data extraction form to extract data from the included studies. Data pertaining to the various attributes of the studies, including the authors’ names, publication year, sample size, research design, and findings, were gathered from every study. The three reviewers independently extracted such data, and any potential disagreement was resolved through consensus and discussions among the reviewers.

Discussion

The initial description of the risk of obesity development in mentally ill persons was done in 1946 by Nicholson [46] who reported psychoneurosis and emotional tensions as being directly linked to obesity [46]. As of the time, several studies focusing on the existing correlations between overweight and obesity and mental health disorders have been conducted with a focus on the extant bidirectional correlations [47]. Additionally, mentally ill persons have indicated a three-fold increment in the risk of developing obesity. On the contrary, studies have suggested that obese persons have a 30% to 70% increased risk of developing a mental health disorder [48]. Further, a survey conducted in North America disclosed that approximately 80% of the 10,000 participants with mental disorders (bipolar, schizophrenia, and depression) were either obese or overweight [48]. As a result of such findings, the rationale underlying the increased number of mentally ill persons living with obesity and overweight has been widely debated.

Moreover, potential correlations between obesity and mental health disorders have been discussed, with certain studies disclosing that individuals with psychiatric disorders face a higher risk of obesity development [49-51] and that those with mental health disorders are highly prone to be overweight or obese compared to the general population [52]. While obesity has been directly linked to impaired mental health, depression, and low quality of life, co-occurrence is more likely to occur in instances where mentally ill persons also have physical impairments. That is, the correlations between mental health disorders and obesity are stronger at later life stages than during earlier stages and in middle-aged persons [53].

Several studies have further attributed the weight gain and development of obesity to the multifaceted interactions between environmental, genetic, and disease-intrinsic aspects and antipsychotic medication side effects [54]. Additional studies have mainly attributed the weight gain and obesity development to the adverse events linked to the use of antipsychotics [55,56]. In this regard, a general assumption has emerged to portray weight gain and obesity as being the adverse side effects of antipsychotic medications, which arise from several mechanisms, including hormone neurotransmission, which includes epinephrine, serotonin, and histamine [57]. Antipsychotics have active agents that work by acting on the neural pathways through modulations of their various activities, and the resultant weight gain and development of obesity might be attributable to the blocking of specific receptors responsible for the regulation of body weight and appetite [55]. According to Volpato et al., antipsychotics and antidepressants may actively increase BMI. They might additionally increase the risk of cardiovascular disease development, indicating a significant increase in metabolic syndrome risk and BMI scores [57].

Previous studies on obesity and weight gain in mentally ill persons have primarily concentrated on risk factors that include poor dietary habits, smoking, and lack of adequate physical exercise [58,59]. Under such context, the effects of medications in relation to weight gain and obesity development have been considered secondary, even though in recent times, more focus has been placed on antipsychotics and the related mental illness diagnoses, including bipolar disorder and schizophrenia, and have revealed significant weight differences that are reliant on the prescribed antipsychotic medication [18,55,56,60,61]. Still, in their study, Zimmerman et al. disclosed that irrespective of the type of medication, antidepressants, antipsychotics, and mood stabilizers, mentally ill persons within the mental healthcare system were highly likely to gain weight and become obese [61]. This has further been corroborated by studies that have revealed that the mean BMI for individuals with personality disorders diagnoses was within the obesity range [62], as well as studies that have indicated that obesity was correlated to cluster A and cluster B personality disorders [63,64].

Regarding the correlation between obesity and personality disorders, it is noteworthy that several studies have reported the existence of a robust and bidirectional relationship between the two conditions [11,65]. However, the prevalence of personality disorders in obese persons has remained unclear; various large-scale surveys have disclosed higher incidence rates of mental health disorders in obese patients, including binge eating disorders, mood disorders, and personality disorders [66-68]. Thus, personality disorders have not only indicated positive correlations with the increase in BMI but additionally, higher interviewer-rated PD rates of 26% have been reported in severely obese individuals seeking obesity treatments such as bariatric surgery [65]. In concurrence, despite persons with mental health disorders having increased odds of being obese, recent studies have indicated that mentally ill persons with personality disorders are increasingly prone to be obese and overweight [11,65,69]. Thus, a higher risk of obesity development has been reported in persons with personality disorders, and recent clinical studies have revealed the existence of comorbidity between personality disorders, including avoidant and borderline, and binge eating disorders [70]. Further, the 17-year cohort Austrian study conducted by Leutner et al. revealed that obese individuals were approximately one and a half times highly prone to have personality disorders compared to the general population without obesity (OR: 1.56, 95% CI: 1.49-1.64) [69].

Furthermore, above-average obesity prevalence has also been found in individuals with bipolar and schizophrenia, in which antipsychotic drugs are used for treatment, and weight gain remains a crucial concern [18,56,60,61]. Also, weight gain has been acknowledged as a critical side-effect of nearly every antipsychotic medication [8,52]. Although antipsychotics vary with regard to their liability in relation to weight gain, second-generation antipsychotics, including olanzapine and clozapine, have a higher potential to bring about weight gain [14,71-73]. Further, medications that include risperidone, iloperidone, quetiapine, and paliperidone have been linked to a modest risk of inducing weight gain., even as medications such as amisulpride, aripiprazole, ziprasidone, lurasidone, and asenapine have minimal effects with regard to weight gain [18]. Moreover, among first-generation antipsychotics (FGAs), thioridazine and chlorpromazine have been acknowledged to cause severe weight gain as compared to haloperidol [18]. In general, all antipsychotic medications are capable of inducing weight gain, given that all antipsychotic drugs have been linked to significant weight gain following prolonged usage [14,16,71,72]. Nonetheless, important differences have been observed between individuals with regard to their vulnerability to either gain or lose weight and develop obesity while using similar antipsychotics [14,71].

In comparison to antipsychotics, it has been observed that antidepressants induce increasingly mild and modest weight gain, and the dissimilarities with regard to weight gain liability between different antidepressants are minimal [8,74]. In this regard, in MDD patients, the long-term usage of antidepressants and potential polypharmacy of various antidepressants, including SSRIs, tetracyclic mirtazapine, and tricyclic antidepressants, has been linked to weight gain in patients of an average of 2.7 kilograms [75,76]. However, it is noteworthy that the antidepressant’s weight-gain effects differ from one person to another.

In BD patients, mood stabilizers have been acknowledged to have considerable effects on weight gain; however, the level of effects is lower compared to those of antipsychotics [8,77]. Furthermore, studies have reported a high frequency of weight gain in instances of lithium use compared to placebo, with an odds ratio of 1.89, with 77% of patients reporting a 6.3-kilogram average weight gain [78,79]. The observed weight gain following lithium use mainly happens in the initial two years, and the increase in BMI indicates a potential increment in weight [79]. The weight gain has been attributed to aspects that include an increase in appetite, nephrogenic diabetes insipidus, and hypothyroidism, which induces thirst [80]. Also, the use of valproate, an antiepileptic mood stabilizer, has been linked to weight gain in over 50% of patients, with an increase in weight being detectable in two to three months upon initiation [81].

Regarding the effects of the correlations between obesity and mental disorders on public health and clinical practice, it is noteworthy that significant focus has been placed on the correlations between obesity and mental disorders in recent times, owing to the increased highlight of the bidirectional relations between the conditions. The intricate relationship has considerable impacts on public health and clinical practice, which necessitates integrated approaches to prevention and treatment. Thus, regarding the public health implications, it is noteworthy that the co-occurrence of obesity and mental disorders is a significant challenge for global public health systems [82]. The conditions have been acknowledged to contribute to the general disease burden while simultaneously increasing the costs of healthcare owing to the associated comorbidities and chronic nature. Moreover, obesity has been associated with the development of chronic health problems that include stroke, type 2 diabetes, certain types of cancer, and heart disease, which are known to have an impact on available healthcare resources and the cost of care for the patients and their families [83].

Existing literature indicates that the incidence of obesity in persons with mental disorders has progressively increased at rates comparable to or even greater than the general population [84]. Moreover, the direction of the relationship between obesity and mental disorders, particularly depression, has been linked to several factors, including age, despite the preliminary level of evidence indicating the causal relationships [85]. For mental disorders, such as schizophrenia, several possible mechanisms have been linked to the increased incidence rates of obesity reported in such populations, despite the appearance of antipsychotic medications as a key contributor [84,85]. Therefore, tackling the dual obesity and mental disorders burden requires comprehensive public health strategies and approaches that mainly target obesity and mental disorders.

The different preventive measures need to prioritize early interventions while also stressing the significance of mental health awareness and the adoption of healthier lifestyle choices from younger ages [86]. Further, the public health campaigns that aim at reducing obesity and mental health-related stigma may promote the seeking of help devoid of fear of being judged. The integration of mental health screening practices into existing obesity prevention programs is also likely to assist in the identification of at-risk persons and offer necessary timely support [86,87]. Additionally, policy-level interventions are important, given that regulations that promote the development of healthy food milieus, including restriction on the marketing of unhealthy foods to young children and enhancing the availability of affordable nutrition alternatives, will aid in tackling the environmental aspects that contribute to obesity development [87]. Similarly, increasing funds for obesity and mental health programs and ascertaining the accessibility of such funds will aid in ensuring the effective management of psychological aspects associated with obesity [88].

The observed correlations between obesity and mental disorders have several implications for clinical practice. Thus, within the clinical practice contexts, the correlations between obesity and mental disorders necessitate the development of effective multidisciplinary treatment approaches [84,86]. In this regard, healthcare providers must take on holistic perspectives, acknowledging the social and psychological dimensions of obesity as opposed to solely concentrating on weight reduction. The clinical practice should integrate regular mental health evaluation into obesity management programs. Depression, eating disorders, and anxiety screening tools should also be utilized, as they may aid clinicians in the identification of the underlying mental health problems that are likely to hinder effective weight management [89]. Further, behavioral therapies, including cognitive behavioral therapy (CBT), have indicated significant efficiency in tackling obesity and correlated psychological health conditions through the therapy’s target of maladaptive behaviors and thought patterns [89].

Consequently, pharmacological interventions have additionally been acknowledged to play a major role in the treatment of obesity and associated mental disorders. For instance, specific medications that include selective serotonin reuptake inhibitors (SSRIs) have been effectively utilized in treating depressive symptoms, and have further been acknowledged to indirectly contribute to weight management through improvement of individual motivation and mood [90]. Nevertheless, clinicians have to be cautious of possible side effects, given that several psychotropic medications have been acknowledged to bring about weight gain. Lastly, fostering a non-judgmental and supportive clinical milieu is vital. Thus, tackling existing weight biases among clinicians and other healthcare professionals is likely to enhance patient care outcomes through the creation of a space in which individuals develop a sense of being understood and respected [90].

Strengths and limitations of this study

Among this study's major strengths are its robust and effective research design and methodology, which enabled the identification of appropriate and high-quality studies for inclusion. Moreover, the included studies have been drawn from various parts of the globe, making the findings of the present research increasingly generalizable to diverse populations. However, the study also has some limitations, including the view that some of the analyses of studies included were associational, making it difficult to arrive at definitive conclusions, particularly with regard to causality. Additionally, the correlations between obesity and different mental disorders are prone to be influenced by other confounding factors and interacting variables that might not have been assessed in this study, including socioeconomic status, dietary and sleep patterns, genetics, and environmental and psychosocial factors. Finally, the dearth of epidemiologically sound cohort studies is another major limitation of the present study, given that the majority of studies included were observational cross-sectional. This has the effect of limiting the level of evidence suggested by this study.

## Conclusions

Globally, obesity remains a chronic medical condition and a major public health problem, which also has a multifactorial etiology. Thus, obesity has been linked to several mental health and physical health conditions and negatively affects the quality of life. Obesity has a multifaceted relationship with mental health and is considered a stress factor given that it adversely impacts mental health in addition to being a major clinical feature in most psychiatric conditions. Obesity has also been found to be a widespread side effect of medications employed in the treatment of various mental health disorders. Nevertheless, a number of lifestyle and biological aspects contribute to the development of mental health disorders and obesity in individuals. For instance, obesity treatment interventions are often multidisciplinary and entail divergent personalized approaches that go beyond physical exercises and diets to include psychological treatments. Comparatively, a larger proportion of studies on obesity and mental health disorders have focused on anxiety and depression, with a limited number of studies focusing on the other diagnoses; however, significant growth of the evidence base has been observed in recent years. In particular, more studies focus on the bi-directional correlations and influence of obesity and mental health. Evidence related to the effectiveness of psychotherapeutic interventions remains inadequate, indicating the need for an increased number of trials, particularly on obese, mentally ill persons.
